# Tunable Polarity Carbon Fibers, a Holistic Approach to Environmental Protection

**DOI:** 10.3390/molecules23051026

**Published:** 2018-04-27

**Authors:** M. Teresa García-Valverde, Carlos A. Ledesma-Escobar, Rafael Lucena, Soledad Cárdenas

**Affiliations:** 1 Grupo FQM-215, Departamento de Química Analítica, Instituto Universitario de Investigación en Química Fina y Nanoquímica (IUIQFN), Universidad de Córdoba, Campus de Rabanales, Edificio Marie Curie, E-14071 Córdoba, Spain; q72gavam@uco.es (M.T.G.-V.); scardenas@uco.es (S.C.); 2 Grupo FQM-227, Departamento de Química Analítica, Instituto Universitario de Investigación en Química Fina y Nanoquímica (IUIQFN), Universidad de Córdoba, Campus de Rabanales, Edificio Marie Curie, E-14071 Córdoba, Spain; car_ledesma@yahoo.com.mx

**Keywords:** carbon fibers, polarity, sorption, analysis

## Abstract

The pollution of environmental resources is an issue of social concern worldwide. Chemistry is essential for the design of decontamination strategies and analytical approaches to detect and monitor the contamination. Sorptive materials are usually required in both approaches and green synthesis should be used to minimize their own environmental impact. Carbon fibers (CFs) obtained by the pyrolysis of natural cellulose-rich materials fulfill these requirements. In this article, thirty CFs obtained under different conditions are chemically characterized and their sorption ability towards selected pollutants, covering a wide range of polarity, is evaluated. This study provides more profound knowledge related to the polarity of these materials, their interactions with chemical substances and allows the prediction of more appropriate materials (pyrolysis temperature and time) in order to remove the given pollutant. Furthermore, the use of CFs as sorptive materials for the extraction of contaminants from water samples to assist with their instrumental detection is outlined. In this sense, the use of CFs and gas chromatography with mass spectrometric detection allows the detection of selected pollutants in the low ng/mL range. Thus, this article provides an integrated approach to the potential of CFs for environmental protection.

## 1. Introduction

Economic development involves the exploitation of natural resources and intensive industrial activity, which produce environmental contamination as undesired collateral damage [1]. Water pollution has become a social concern due to its deleterious effect on human health and the environment. In response to this environmental awareness, the European Commission has defined water as a heritage that must be protected, defended and treated as such [2]. Chemistry plays a crucial role in the remediation of pollution by providing the strategies for removing pollutants from natural water resources. In the last decades, photocatalysis [3,4] and sorption [5,6,7] approaches have emerged as tools in this context, allowing the degradation or isolation of harmful compounds from different environmental compartments.

Sorption strategies require high-capacity and low-cost materials [8] that are able to interact with the target pollutants. Although synthetic materials, such as carbon nanotubes sponges [9], fulfill the first requirement, their prices are not always competitive. Natural materials and industrial by-products emerge as a better option in this context and they involve a greener vision: the use of sustainable methods and materials to remediate pollution. Natural materials cover a wide range of products, including winter melon [10], avocado seeds [11,12], garlic [13] and orange peels [14,15]. Industrial by-products [16] may have different origins, such as scrap tires [17] and carbon that is derived from biomass gasification [18].

The natural products used in the environmental remediation are cellulose-rich materials. Cellulose is an abundant, sustainable and environmental friendly precursor that can be obtained both in bottom-up [19] (using bacteria) or top-down (using raw natural resources) approaches. It is a hydrophilic biopolymer that can interact with ionic species, such as metals and certain dyes, due to its native or enhanced ion-exchange capacity [20]. Its polar nature hinders its interaction with hydrophobic substances and thus, the chemical modification [21] of cellulose is necessary in order to broaden the application scope. The derivatization of the superficial functional groups of cellulose [22], the immobilization of carbon nanoparticles [23] on the cellulosic fibers and the use of cold-plasma derivatization [24] are some of the reported strategies. However, these approaches involve the use of chemical reagents in multi-step synthesis workflows, thus reducing their green nature. Pyrolysis, which is based on heating the cellulosic material at a high temperature in an inert atmosphere (N_2_ or Ar), is a greener and solventless alternative [25]. If the pyrolysis temperature is appropriately selected, the resulting material maintains its fibrous structure [26], although the fibers become thinner [27], which allows for easier handling of the resulting product. The modifications of this general workflow have been reported, including the pre-treatment of the fibers [28], the final activation of the carbon heating the fibers in a gas flow containing water [29] and the final transformation of the fibers in a sorptive powder [30].

The polarity of the fibers is critical for defining the type and intensity of the interactions with the target pollutants. As demonstrated in the synthesis of carbonaceous materials, this polarity can be fine-tuned by adjusting the pyrolysis temperature [31,32]. This paper studies how the polarity of the carbon fibers can be modified according to the pyrolysis conditions (temperature and time). The effect of this treatment on the fibers’ sorption ability towards different compounds covering a wide range of octanol–water partition-coefficient (log P), is deeply studied. This ability, which is crucial for environmental remediation, is of interest in the chemical analysis of environmental compartments. These analyses that allow the detection of pollution and the monitoring of the remediation processes usually require an extraction of the target compounds from the sample matrix before their instrumental determination [33]. The sensitivity and selectivity of these measurements rely on the proper selection of the sorbent, with the polarity being a critical parameter. In summary, this article provides an integrated vision of the use of carbon fibers in environmental protection, including chemical remediation and pollution monitoring.

## 2. Materials and Methods

### 2.1. Reagents

All the reagents were of analytical grade or better. Unless otherwise specified, they were purchased from Sigma Aldrich (Madrid, Spain). The stock standard solutions of the compounds (cyclohexane, heptane, octane, heptanal, octanal, decanal, benzaldehyde and methylbenzaldehyde) were prepared in methanol (Panreac, Barcelona, Spain) at a concentration of 0.05 g/L and stored at 4 °C in the dark. The working standards were prepared daily by the dilution of the stock solutions in Milli-Q water from Merck/Millipore (Molsheim, France). 

Commercial cotton was used as the raw material for the synthesis of the carbon fibers (CFs). Cotton mainly consists of cellulose (in the range of 80–90%) and other secondary components, such as water, fats and inorganic ashes. Ar (Carburos metálicos, Barcelona, Spain) was used as the inert gas during the pyrolysis.

### 2.2. Synthesis of Carbon Fibers

CFs were synthesized in a simple, quick and cheap process [34]. This synthetic approach also provides a good reproducibility (relative standard deviations that were lower than 2.5%), with similar carbon content of the carbon fibers from different batches. In this sense, 4 g of raw cotton was introduced into a stainless-steel tube with a hermetic seal. The tube was purged with an Ar stream for 20 min to remove the oxygen. Finally, the tubes were sealed and heated at a pre-determined temperature and time. Thirty different materials were synthesized using different temperatures (200, 300, 400, 500, 600 and 700 °C) for different periods of time (0.5, 1, 2, 3 and 4 h).

### 2.3. Characterization of Carbon Fibers

The synthesized fibers were characterized using scanning electron microscopy (SEM), elemental analysis and infrared spectroscopy. The SEM images were obtained using a JEOL JSM 6300 microscope, while the carbon, nitrogen and oxygen content of the fibers were determined in a EuroVector Elemental Analyser EA3000 (EuroVector SpA, Milan, Italy). Both micrographs and elemental analysis were conducted at the Central Service for Research Support (SCAI) of the University of Córdoba.

The infrared measurements were performed in a Bruker Tensor37 infrared (IR) spectrometer, which was equipped with a diamond attenuated total reflection (ATR) cell with a circular surface of 3-mm diameter and three internal reflections. A Deuterated Triglycine Sulfate detector was used for the acquisition of the spectra. The spectra were collected in the wavelength range from 4000 to 500 cm^−1^ at a resolution of 4 cm^−1^ with 64 coadded scans each. Data collection was conducted using the OPUS software (Bruker, Ettligen, Germany).

### 2.4. Sorption Studies of the Carbon Fibers

The sorption ability of the synthesized materials towards different substances with log P of 1.64–5.09 (viz.: benzaldehyde, methylbenzaldehyde, heptanal, octanal, cyclohexane, decanal, heptane and octane) was evaluated. For this purpose, 20 mg of each material were dispersed and incubated (during 30 min) in 5 mL of the aqueous standards of the model substances. After the incubation time, 1 mL of the headspace (HS) of the vial was analyzed by gas chromatography-mass spectrometry (GC-MS). The concentration of the compounds in the HS is proportional to their free concentration in the aqueous phase. Therefore, a strong retention of the compound by the carbon fibers necessarily involves a reduction in its concentration in the vial headspace [35]. A calibration curve prepared with standards in the absence of CFs allows the quantitation of the compounds. The HS-GC-MS conditions are explained in the Supplementary Information.

To present an integrated vision, the potential of CFs as sorbents for analytical purposes was also examined. A simple device (shown in Figure S1) is employed for the extraction of the samples, which consisted of a glass syringe where the CFs are located. A defined volume of water sample spiked with the target pollutants is passed through the system. Due to the CF–pollutant interactions, the pollutants are retained and separated from the sample matrix. This interaction is broken using an appropriate solvent (chloroform), and the chloroformic extract is finally injected in the GC-MS equipment for analysis. The settings of the GC-MS analysis are also presented in Supplementary Information.

## 3. Results and Discussion

**Figure 1:**
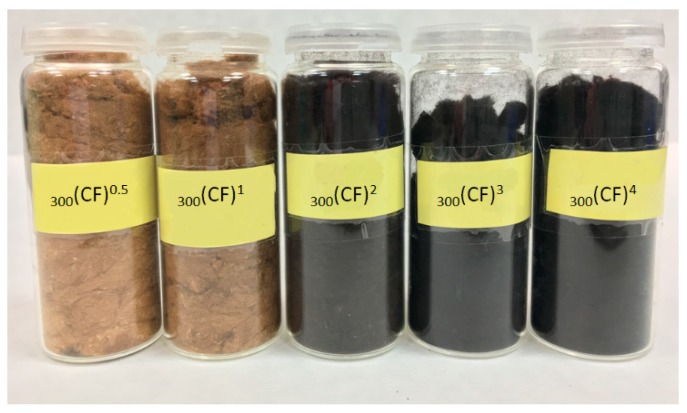
Pictures of the CFs synthesized at 300 °C.

Thirty different CFs were synthesized by heating commercial cotton at several temperatures (200, 300, 400, 500, 600 and 700 °C) for different periods of times (0.5, 1, 2, 3 and 4 h) under an inert atmosphere of Ar. For simplicity, the carbon fibers will henceforth be denoted as _T_(CF)^t^, where T and t are the pyrolysis temperature and time, respectively. A preliminary evaluation of the fibers showed a progressive darkening of the material with increasing temperature and a longer period of time. This progressive darkening of the material can be seen in Figure 1 for the materials synthesized at 300 °C due to the carbon formation.

**Figure 2:**
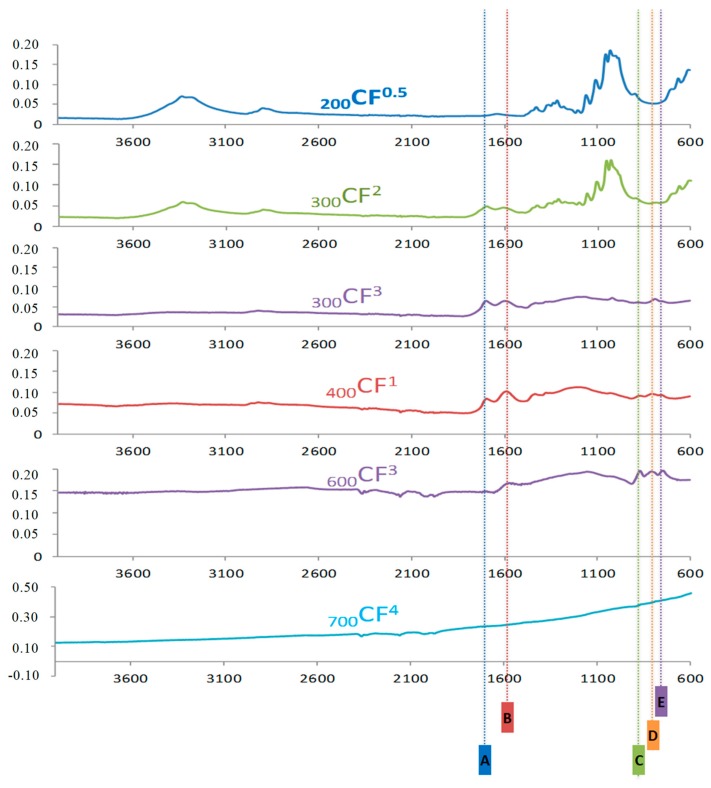
ATR-IR spectra of six selected materials. Five absorption bands are identified, which are namely at: (A) 1706 cm^−1^, (B) 1560 cm^−1^, (C) 879 cm^−1^, (D) 819 cm^−1^ and (E) 758 cm^−1^. The spectra show the absorbance and the wavelength in the y-axis and x-axis, respectively.

The infrared spectra of all the materials were acquired under the ATR mode. For simplicity, Figure 2 only focuses the discussion on six representative spectra, which allow the monitoring of the pyrolysis of the cotton fibers. This pyrolysis is a complex process, which is still under discussion as it involves multi-phase reactions [25,36]. The spectrum of _200_CF^0.5^ is very similar to that obtained for cellulose, which indicates that these mild conditions cannot have a dramatic effect on the chemical composition of the raw material. The rich spectrum of cellulose begins to be affected by more aggressive conditions. As it can be observed in the spectrum of _300_CF^2^, the cellulosic bands begin to decline, while two new bands at 1706 and 1560 cm^−1^ slightly appear in the spectrum. The intensity of these bands increases after a longer period of time as it is observable in the spectrum of _300_CF^3^. The band at 1706 cm^−1^ is assigned to a ketone group that is formed due to a keto-enol tautomerism after the loss of a water molecule. The band at 1560 cm^−1^ is assigned to a C=C (stretching) in a cyclic structure. The relative intensities of the bands at 1706 and 1560 cm^−1^ vary in _400_CF^1^, indicating that the carbonyl group is finally lost. As temperature and time increase, two additional bands at 879 and 758 cm^−1^ appear (see _600_CF^3^). The bands at 879, 806 and 758 cm^−1^ are assigned to the aromatic C-H out-of-plane bending vibrations [37]. At 700 °C, the spectra do not show clear absorption bands, while the baseline increases dramatically (see _700_CF^4^). This effect is usually observed when the high-carbon content materials are analyzed by ATR-IR.

This discussion can be enriched by considering the elemental analysis results of the materials, which are included in Table S1. According to these results, all the materials synthesized at 200 °C, _300_CF^0.5^ and _300_CF^1^ present a chemical composition similar to that provided by cellulose (C_6_H_10_O_5_), indicating a reduced pyrolysis under these conditions. At higher temperatures and longer periods of time, a continuous increase in the carbon content is observed, while a reduction in hydrogen and oxygen is noted. It is well known that the polarity of a given material can be defined according to its C, N and O content. In fact, some authors have defined an arbitrary polarity index (PI) according to the equation PI = (O + N)/C, which can be simplified in our study as nitrogen is not present [38]. Therefore, the polarity index of the materials decreases from 1.1 (value for raw cellulose) to 0.07 during the pyrolysis.

**Figure 3:**
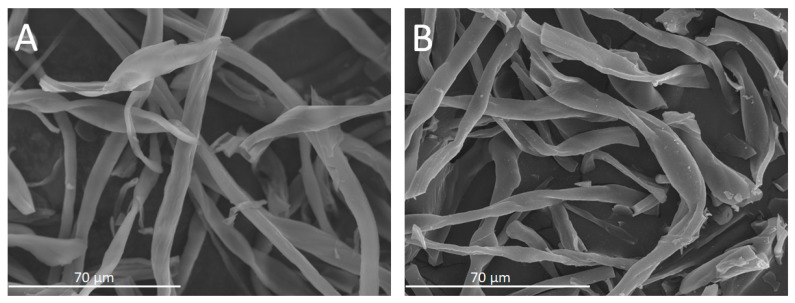
SEM micrographs of (**A**) _400_CF^2^ and (**B**) _700_CF^4^.

The maximum carbon content is achieved in the materials synthesized at 600 and 700 °C. In both cases, hydrogen and oxygen are still present and their respective functional groups may be involved in maintaining the fibrous structure. As observed in Figure 3, the SEM images for _400_CF^2^ and _700_CF^4^ (both materials are selected as the representatives of moderate and hard conditions, respectively) showed a fibrous structure. However, _700_CF^4^ demonstrates that more damage was found in the structure.

The sorption ability of the materials was studied by HS-GC-MS using several compounds of different polarity as the model pollutants. The differences in the extraction capability among materials were evaluated by analysis of variance (ANOVA), pairwise mean comparison (Tukey HSD) and partial least squares-discriminant analysis (PLS-DA). The extraction capability of all developed materials was evaluated in triplicate and the retention efficiency of all studied compounds was calculated. The resultant matrix (90×08) was transformed by Log2 to reduce the data heteroscedasticity, before being scaled by an autoscaling method to ensure that all compounds are equally important in the analysis irrespective of their concentration. Both ANOVA and PLS-DA were applied by using MetaboAnalyst 3.0 (www.metaboanalyst.ca). Additionally, to determine the suited synthesis conditions that allow the material to extract all studied compounds, the obtained results were fitted into a second-order polynomial model (Equation (1)) and analyzed by response surface methodology:

Y_i_ = β_0_ + β_m_X_m_ + β_n_X_n_ + β_mm_X_m_^2^ + β_mn_X_m_X_n_ + β_nn_X_n_^2^ (1)

Furthermore, a desirability function approach (Equation (2)) was used to predict the suited synthesis conditions to maximize: (i) the retention of all eight compounds; and (ii) the retention of either polar or low-polar compounds.

(2) $$D=(d_{i}\left(Y_{i}\right)\ldots {\left(d_{n}\left(Y_{n}\right)\right)}^{\frac{1}{n}},$$

where $d_{i}\left(Y_{i}\right)=\frac{Y_{i}-Y_{\min}}{Y_{\max}-Y_{\min}}$.

For each response Y_i_ (X), a desirability function d_i_ (Y_i_) = 1 represents the completely desirable value or the best value obtained by the surface response model. The optimization steps were developed by Statgraphics Centurion XVI (Stat-Point Technologies 2011, Golden Valley, MN, USA).

**Figure 4:**
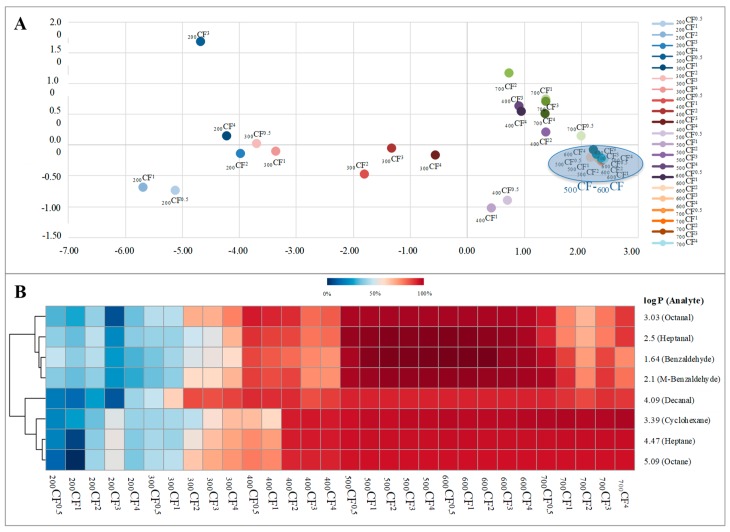
Scores of the PLS-DA (**A**) and heatmap (**B**) comparing the averaged retention capability of the eight synthesized materials.

A supervised analysis by PLS-DA was used to discriminate the 30 materials. The averaged retention efficiency of all eight studied compounds for each treatment was used to create the data matrix (30×08) used in the multivariate analysis. The two-dimensional PLS-DA explained 95.6% (Component1 = 90.5% and Component2 = 5.1%) of the total variability. The plot of the scores (Figure 4A) shows a clear discrimination among materials and reveals that the distance between scores in the plot was shorter between the materials synthesized at both 500 °C and 600 °C, regardless of the time. Considering only the materials obtained at these temperatures, the PLS-DA exhibits greater similarities among the materials obtained at 500 °C for a longer period of time (over 2 h) and those obtained at 600 °C for a shorter period of time (shorter than 2 h). Figure S2 zooms in and focuses on the 500–600 °C region in the PLS-DA. Additionally, the heatmap (Figure 4B) revealed that the materials synthesized at both 500 °C and 600 °C possess the highest retention capacity of all studied compounds. Furthermore, the heatmap demonstrated that the differences in the retention capacity of the materials increased when the differences in log P among analytes were higher. To determine the suited synthesis conditions for the retention of each material, the results were analyzed by response surface methodology (Equation (1)).

The ANOVA test revealed a significant (*p* < 0.05) effect of the temperature on the extraction capability for all synthesized materials, while the time was significant (*p* < 0.05) only for the most polar compounds (benzaldehyde, methylbenzaldehyde, heptanal and octanol, with log P < 3.39). In general, the results indicate that more aggressive conditions are required to retain the most hydrophobic compounds. This fact is consistent with ATR-IR and elemental analysis, since the strong conditions induce a modification in the chemical composition of the raw material, reducing its polarity. In this sense, the materials heated at 577 °C and 588 °C for 0.5 h are more suitable for retaining hydrophilic compounds, such as benzaldehyde (log P = 1.64) and methylbenzaldehyde (log P = 2.10), whereas the materials treated at higher temperatures and times (t > 2.6 h and T > 653 °C) are more appropriate for retaining hydrophobic compounds, such as heptane (log P = 4.47) and octane (log P = 5.09). Figure S3 shows the optimum synthesis conditions for the extraction of all components individually and the effect of the time and temperature on the retention capability. Furthermore, the observed differences in the studied synthesis conditions as a function of the polarity were confirmed by the results obtained by multiple objective optimization. According to this analysis, the suited conditions for the simultaneous retention of the most hydrophilic compounds (benzaldehyde, methylbenzaldehyde, heptanal and octanal) were 2.5 h and 637 °C. For the most hydrophobic compounds (cyclohexane, decanal, heptane and octane), 0.5 h and 561 °C are required (Supplementary Figure S4A,B). Finally, the suited conditions for maximizing the simultaneous extraction of the eight studied compounds were 0.5 h and 607 °C, which provide over 92% of the maximum retention of all studied compounds (Supplementary Figure S4C).

**Figure 5:**
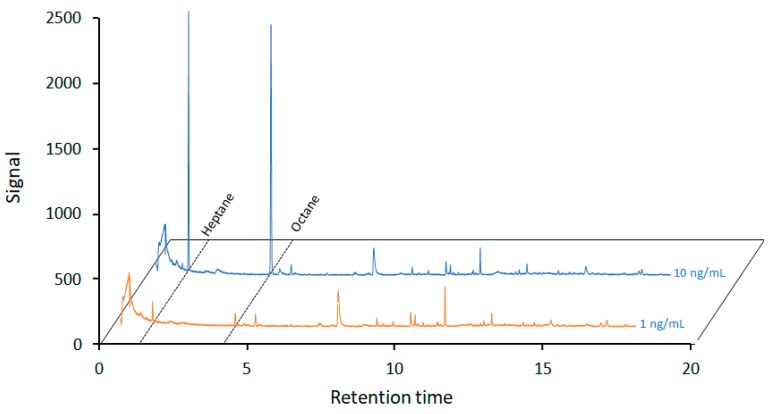
Chromatograms of a water sample spiked with heptane and octane at 10 and 1 ng/mL, which were extracted with the _600_CF^0.5^.

Once a relationship between the polarity of the CFs and the analytes was demonstrated, the potential of CFs for the analysis of pollutants in water samples was examined. In this case, the CFs are applied using the device described in Supplementary Information. For simplicity, heptane and octane were selected as model analytes. A total of 250 mL of the water sample was spiked with both analytes at concentration levels of 10 ng/mL and 1 ng/mL. This volume was processed at a high flow rate (up to 12.5 mL/min) using _600_CF^0.5^ (material selected by a compromise). After the extraction, the pollutants were recovered using chloroform, before 4 µL of these extracts were analyzed by GC-MS. The results demonstrated the ability of the CFs to retain and preconcentrate the analytes from the samples (Figure 5). The peaks corresponding to heptane (retention time, 3.19 min) and octane (retention time, 5.98 min) are clearly identified. The heptane peak presents a signal-to-noise ratio of 580 and 49.8 at 10 and 1 ng/mL, respectively. The octane peak presents a signal-to-noise ratio of 710 and 39.4 at 10 and 1 ng/mL, respectively. These data are also presented in Figure S5.

## 4. Conclusions

This article provides a holistic approach to the use of CFs in environmental protection. Their sorption properties can be exploited for the isolation of pollutants from water for both remediation and analytical purposes. The polarity of the CFs, which is crucial for defining their interactions with the molecules of a given contaminant, can be selected by modifying the temperature and time of pyrolysis. When combined with the proper mathematical model, this aspect allows us to select the best synthetic conditions with consideration of the polarity of the target compound. Furthermore, this allows us to minimize the energy required in the pyrolysis as the CFs can be prepared at the softer conditions (minimum temperature and time) that are required to assess a good performance.

As it has been demonstrated, the pyrolysis conditions have an evident influence on the sorptive properties of the CFs. The more polar compounds among those studied are extracted with CFs that were synthesized at moderate conditions, while more hydrophobic substances require CFs that were obtained at more aggressive conditions.

Furthermore, the sorption can be exploited in environmental monitoring as a part of the sample treatment before instrumental analysis. Working at non-optimized conditions, the use of CFs as sorbents under a solid phase extraction format allows the detection of heptane and octane (selected as the model analytes to show the potential of this approach) at the low ng/mL range.

Further studies will focus on the effect of polarity on the sorption kinetics since the wettability of the material plays a key role in the extraction. It would be also interesting to study how these CFs work in a hydrophobic media. In such conditions, the more polar CFs could be used to recover valuable by-products from industrial wastes (e.g., olive oil production).

Two additional aspects that are essential for the in-field applications of these fibers will be considered in further research. On the one hand, the economic costs associated with the synthesis (which involves the heating of the natural material at high temperatures) must be evaluated. On the other hand, the reproducibility of the synthesis, which was only evaluated in this article at the lab-scale, should be considered again at the industrial scale. This batch-to-batch reproducibility will be especially important for analytical applications where the sorption capacity influences the sensitivity of the determination.

## Supplementary Materials

Supplementary materials are available online. Figure S1: (A) 50-mL glass syringe containing a paper filter for CFs confinement; (B) Last milliliters of sample passing through the confined CFs. Figure S2: Scores of the PLS-DA from all materials synthesized at 500 or 600 °C. Figure S3: Surface response plot, optimum conditions and effects of the time and temperature on the synthesis of the materials for individual extraction of all studied compounds. Figure S4: Surface response plot and optimum conditions for the simultaneous extraction of the: (A) less polar, (B) most polar, and (C) total studied compounds. Figure S5: Detail of the chromatographic peaks obtained for heptane and octane at 10 ng/mL and 1 ng/mL. The signal-to-noise ratio (S/N) is also indicated. Table S1: Elemental analysis of the carbon fibers.

## References

[1] Stern D.I. (2004). The Rise and Fall of the Environmental Kuznets Curve. World Dev..

[2] (2000). European Community Directive 2000/60/EC of the European Parliament and of the Council of 23 October 2000 establishing a framework for Community action in the field of water policy. Off. J. Eur. Parliam..

[3] Pelaez M., Nolan N.T., Pillai S.C., Seery M.K., Falaras P., Kontos A.G., Dunlop P.S.M., Hamilton J.W.J., Byrne J.A., O’Shea K. (2012). A review on the visible light active titanium dioxide photocatalysts for environmental applications. Appl. Catal. B Environ..

[4] Singh S., Mahalingam H., Singh P.K. (2013). Polymer-supported titanium dioxide photocatalysts for environmental remediation: A review. Appl. Catal. A Gen..

[5] Kemp K.C., Seema H., Saleh M., Le N.H., Mahesh K., Chandra V., Kim K.S. (2013). Environmental applications using graphene composites: Water remediation and gas adsorption. Nanoscale.

[6] Maleki H. (2016). Recent advances in aerogels for environmental remediation applications: A review. Chem. Eng. J..

[7] Khin M.M., Nair A.S., Babu V.J., Murugan R., Ramakrishna S. (2012). A review on nanomaterials for environmental remediation. Energy Environ. Sci..

[8] Björklund K., Li L. (2015). Evaluation of low-cost materials for sorption of hydrophobic organic pollutants in stormwater. J. Environ. Manag..

[9] Gui X., Wei J., Wang K., Cao A., Zhu H., Jia Y., Shu Q., Wu D. (2010). Carbon nanotube sponges. Adv. Mater..

[10] Li Y.Q., Samad Y.A., Polychronopoulou K., Alhassan S.M., Liao K. (2014). Carbon aerogel from winter melon for highly efficient and recyclable oils and organic solvents absorption. ACS Sustain. Chem. Eng..

[11] Elizalde-González M.P., Mattusch J., Peláez-Cid A.A., Wennrich R. (2007). Characterization of adsorbent materials prepared from avocado kernel seeds: Natural, activated and carbonized forms. J. Anal. Appl. Pyrolysis.

[12] Rodrigues L.A., da Silva M.L.C.P., Alvarez-Mendes M.O., dos Reis Coutinho A., Thim G.P. (2011). Phenol removal from aqueous solution by activated carbon produced from avocado kernel seeds. Chem. Eng. J..

[13] Hameed B.H., Ahmad A.A. (2009). Batch adsorption of methylene blue from aqueous solution by garlic peel, an agricultural waste biomass. J. Hazard. Mater..

[14] Namasivayam C., Muniasamy N., Gayatri K., Rani M., Ranganathan K. (1996). Removal of dyes from aqueous solutions by cellulosic waste orange peel. Bioresour. Technol..

[15] Munagapati V.S., Kim D.S. (2016). Adsorption of anionic azo dye Congo Red from aqueous solution by Cationic Modified Orange Peel Powder. J. Mol. Liq..

[16] Wendling L.A., Douglas G.B., Coleman S., Yuan Z. (2013). Nutrient and dissolved organic carbon removal from natural waters using industrial by-products. Sci. Total Environ..

[17] Li L., Liu S., Zhu T. (2010). Application of activated carbon derived from scrap tires for adsorption of Rhodamine B. J. Environ. Sci..

[18] Maneerung T., Liew J., Dai Y., Kawi S., Chong C., Wang C.H. (2016). Activated carbon derived from carbon residue from biomass gasification and its application for dye adsorption: Kinetics, isotherms and thermodynamic studies. Bioresour. Technol..

[19] Lin S.P., Loira Calvar I., Catchmark J.M., Liu J.R., Demirci A., Cheng K.C. (2013). Biosynthesis, production and applications of bacterial cellulose. Cellulose.

[20] O’Connell D.W., Birkinshaw C., O’Dwyer T.F. (2008). Heavy metal adsorbents prepared from the modification of cellulose: A review. Bioresour. Technol..

[21] Hokkanen S., Bhatnagar A., Sillanpää M. (2016). A review on modification methods to cellulose-based adsorbents to improve adsorption capacity. Water Res..

[22] Tserki V., Zafeiropoulos N.E., Simon F., Panayiotou C. (2005). A study of the effect of acetylation and propionylation surface treatments on natural fibres. Compos. Part A Appl. Sci. Manuf..

[23] Zhang C., Zhang R.Z., Ma Y.Q., Guan W.B., Wu X.L., Liu X., Li H., Du Y.L., Pan C.P. (2015). Preparation of cellulose/graphene composite and its applications for triazine pesticides adsorption from water. ACS Sustain. Chem. Eng..

[24] Lin R., Li A., Zheng T., Lu L., Cao Y. (2015). Hydrophobic and flexible cellulose aerogel as an efficient, green and reusable oil sorbent. RSC Adv..

[25] Lin Y.C., Cho J., Tompsett G.A., Westmoreland P.R., Huber G.W. (2009). Kinetics and mechanism of cellulose pyrolysis. J. Phys. Chem. C.

[26] Jiao Y., Wan C., Li J. (2016). Synthesis of carbon fiber aerogel from natural bamboo fiber and its application as a green high-efficiency and recyclable adsorbent. Mater. Des..

[27] Bi H., Yin Z., Cao X., Xie X., Tan C., Huang X., Chen B., Chen F., Yang Q., Bu X. (2013). Carbon fiber aerogel made from raw cotton: A novel, efficient and recyclable sorbent for oils and organic solvents. Adv. Mater..

[28] Štefelová J., Slovák V., Siqueira G., Olsson R.T., Tingaut P., Zimmermann T., Sehaqui H. (2017). Drying and Pyrolysis of Cellulose Nanofibers from Wood, Bacteria, and Algae for Char Application in Oil Absorption and Dye Adsorption. ACS Sustain. Chem. Eng..

[29] Li K., Zheng Z., Feng J., Zhang J., Luo X., Zhao G., Huang X. (2009). Adsorption of p-nitroaniline from aqueous solutions onto activated carbon fiber prepared from cotton stalk. J. Hazard. Mater..

[30] Chen H., Wang X., Li J., Wang X. (2015). Cotton derived carbonaceous aerogels for the efficient removal of organic pollutants and heavy metal ions. J. Mater. Chem. A.

[31] Niu H., Wang Y., Zhang X., Meng Z., Cai Y. (2012). Easy synthesis of surface-tunable carbon-encapsulated magnetic nanoparticles: Adsorbents for selective isolation and preconcentration of organic pollutants. ACS Appl. Mater. Interfaces.

[32] Al-Wabel M.I., Al-Omran A., El-Naggar A.H., Nadeem M., Usman A.R.A. (2013). Pyrolysis temperature induced changes in characteristics and chemical composition of biochar produced from conocarpus wastes. Bioresour. Technol..

[33] Souza-Silva É.A., Jiang R., Rodríguez-Lafuente A., Gionfriddo E., Pawliszyn J. (2015). A critical review of the state of the art of solid-phase microextraction of complex matrices I. Environmental analysis. TrAC Trends Anal. Chem..

[34] García-Valverde M.T., Lucena R., Cárdenas S., Valcárcel M. (2016). In-syringe dispersive micro-solid phase extraction using carbon fibres for the determination of chlorophenols in human urine by gas chromatography/mass spectrometry. J. Chromatogr. A.

[35] Carrillo-Carrión C., Lucena R., Cárdenas S., Valcárcel M. (2007). Surfactant-coated carbon nanotubes as pseudophases in liquid-liquid extraction. Analyst.

[36] Şerbǎnescu C. (2014). Kinetic analysis of cellulose pyrolysis: A short review. Chem. Pap..

[37] Budarin V., Clark J.H., Hardy J.J.E., Luque R., Milkowski K., Tavener S.J., Wilson A.J. (2006). Starbons: New starch-derived mesoporous carbonaceous materials with tunable properties. Angew. Chem. Int. Ed..

[38] Xing B., McGill W.B., Dudas M.J. (1994). Sorption of α-naphthol onto organic sorbents varying in polarity and aromaticity. Chemosphere.

