# Supplementary material for: Tunable Polarity Carbon Fibers, a Holistic Approach to Environmental Protection
Source: Molecules. 2018 Apr 27;23(5):1026. doi: 10.3390/molecules23051026 (PMC6102572; doi:10.3390/molecules23051026)
Supplement: Supplementary file 1 [file molecules-23-01026-s001.pdf]

Tunable polarity carbon fibers, a holistic approach to environmental protection

M. Teresa García-Valverde,[a] Carlos A. Ledesma-Escobar,[b] Rafael Lucena,[a*] Soledad Cárdenas[a]

[a] *Departamento de Química Analítica, Instituto Universitario de Investigación en Química Fina y Nanoquímica (IUIQFN), Universidad de Córdoba, Campus de Rabanales, Edificio Marie Curie, E-14071 Córdoba, España. Grupo FQM-215 email: rafael.lucena@uco.es*

[b] *Departamento de Química Analítica, Instituto Universitario de Investigación en Química Fina y Nanoquímica (IUIQFN), Universidad de Córdoba, Campus de Rabanales, Edificio Marie Curie, E-14071 Córdoba, España. Grupo FQM-227.*

5 Figures

1 Table

1. HS-GC-MS analysis.

Headspace analyses were performed on a MPS2 32-space headspace autosampler (Gerstel, Mülhein an der Ruhr, Germany) including a robotic arm and an oven. An automated injector was used for the introduction of the vial headspace into the HP6890 gas chromatograph (Palo Alto, CA, USA) equipped with a HP5973 mass spectrometric detector based on a quadrupole analyzer and an electron multiplier detector. System control and data acquisition were achieved with a HP1701CA MS ChemStation software.

A column split ratio of 1:10 was selected for injection, using Helium (6.0 grade, Air liquid, Seville, Spain) at a flow rate of 1 mL/min as carrier gas. Chromatographic separations were performed on a fused silica HP-5MS capillary column, 30 m × 0.25 mm i.d. coated with 5% diphenyl, 95% dimethyl polysiloxane (film thickness 0.25 μm) from Agilent. The column temperature program was as follows: 5 min at 30 °C, raised up to 130 °C at 10 °C/min keeping this temperature for 5 min.

Electron impact ionization (70 eV) was used for analytes fragmentation. The quadrupole mass spectrometer detector was operated in selected ion monitoring mode, recording the following fragment-ions: 84 (from 2.00 to 3.90 min), 43 (from 3.90 to 9.00 min), 70 (from 9.00 to 10.90 min), 77 (from 10.90 to 11.90 min), 41 (from 11.90 to 12.75), 91 (from 12.75 to 14.00) and finally 57 (from 14.00 min to the end of the chromatogram). The injector, MS source and quadrupole temperatures were kept at 250 °C, 230 °C and 150 °C, respectively. The peak areas were used for quantification of individual analytes.

2. Extraction of the analytes from water samples and subsequent GC-MS analysis

The extraction of the samples can be done in the system depicted in FigureS1. It consists of a 50-mL glass syringe fitted with a filter paper to allow the confinement of the CFs (Figure S1A). The sample is passed through the CFs, as it is indicated in Figure S1B, to promote the interaction with the pollutants. This interaction permits the retention of the pollutants and their separation from the sample matrix. The pollutants are finally eluted with 2 mL of chloroform and 4 µL of this extract is subsequently injected in GC-MS for analysis.

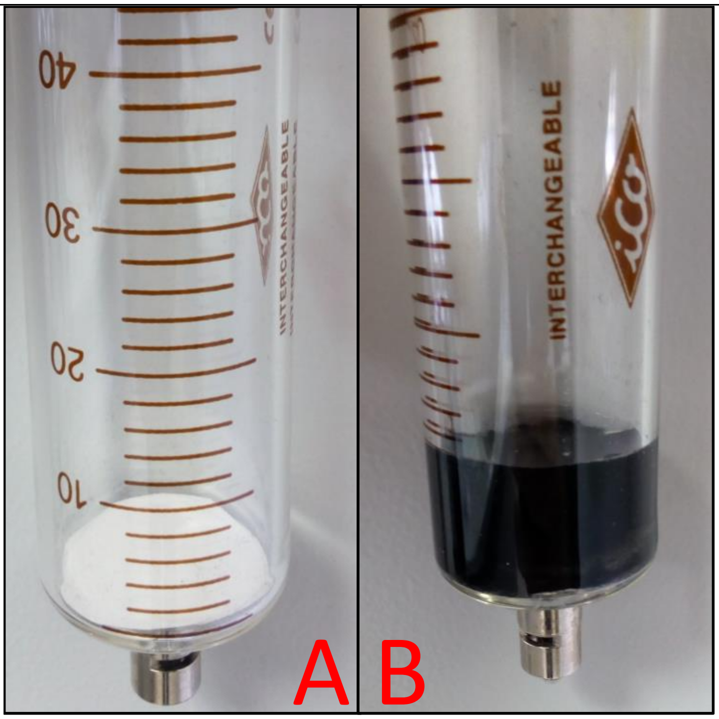

Figure S1. A) 50-mL glass syringe containing a paper filter for CFs confinement; B) Last milliliters of sample passing through the confined CFs

GC-MS analyses were performed on a 7890A Agilent gas chromatograph equipped with ah 5975C MS detector consisting on a quadrupole analyzer and an electron multiplier detector. The conditions for the gas chromatographic/MS analyses were equal to those described for

the HS measurements. However, the fragment ion 57 was selected for the quantification of heptane and octane as this fragment present a better sensitivity/selectivity balance, which is necessary when samples are processed. Also, 4 µL was selected as injection volume.

3. Elemental analysis of the CFs

Table S1. Elemental analysis of the carbon fibers.

Material	%C	%H	%O	Material	%C	%H	%O
$_{200}CF^{0.5}$	43.1	7.3	49.5	$_{500}CF^{0.5}$	82.2	4.3	13.3
$_{200}CF^{1}$	42.8	7.6	49.5	$_{500}CF^{1}$	85.4	4.1	10.3
$_{200}CF^{2}$	43.6	7.0	49.3	$_{500}CF^{2}$	86.0	4.2	9.6
$_{200}CF^{3}$	43.3	7.7	48.9	$_{500}CF^{3}$	87.6	4.1	8.1
$_{200}CF^{4}$	43.3	7.8	48.6	$_{500}CF^{4}$	87.0	4.0	8.9
$_{300}CF^{0.5}$	44.6	7.8	47.6	$_{600}CF^{0.5}$	90.8	3.4	5.6
$_{300}CF^{1}$	45.0	7.8	47.1	$_{600}CF^{1}$	92.1	3.6	4.2
$_{300}CF^{2}$	53.6	6.9	39.4	$_{600}CF^{2}$	91.7	3.4	4.9
$_{300}CF^{3}$	70.2	5.8	23.7	$_{600}CF^{3}$	90.6	3.2	6.3
$_{300}CF^{4}$	69.3	5.7	25.0	$_{600}CF^{4}$	91.4	3.4	5.2
$_{400}CF^{0.5}$	74.2	5.3	20.3	$_{700}CF^{0.5}$	90.8	2.8	6.3
$_{400}CF^{1}$	77.3	5.5	17.0	$_{700}CF^{1}$	90.1	2.6	7.3
$_{400}CF^{2}$	76.9	5.1	17.7	$_{700}CF^{2}$	89.4	2.4	8.2
$_{400}CF^{3}$	79.6	5.2	15.0	$_{700}CF^{3}$	90.0	2.2	7.9
$_{400}CF^{4}$	79.9	5.2	14.6	$_{700}CF^{4}$	91.0	2.1	6.8

4. Sorption ability of CFs

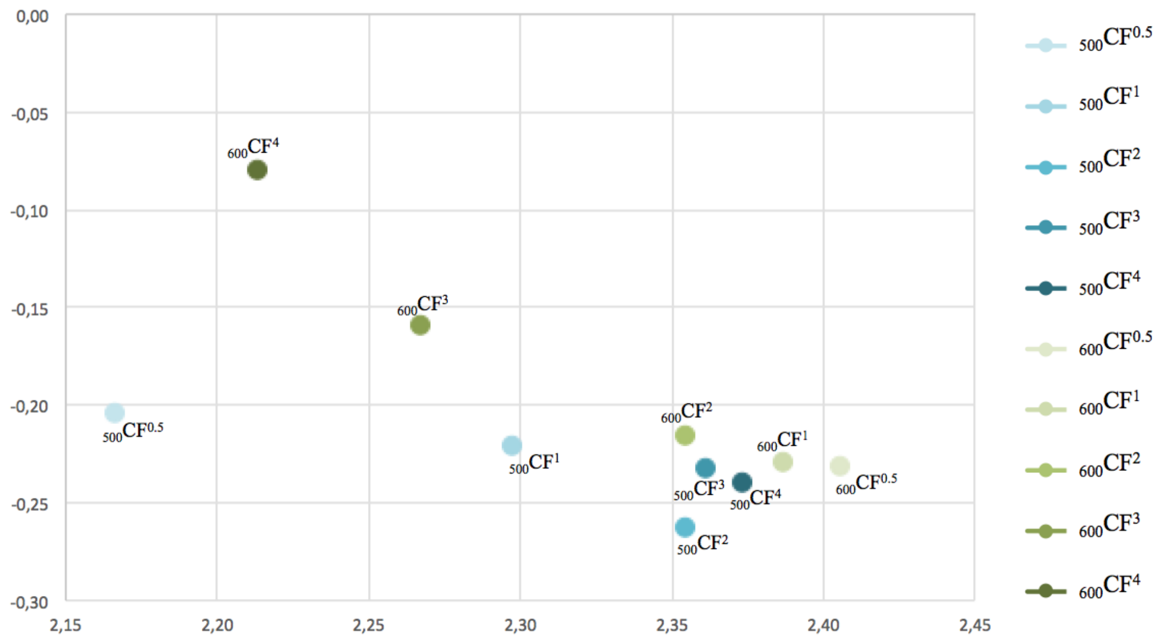

Figure S2. Scores of the PLS-DA from all materials synthesised at 500 or 600 °C.

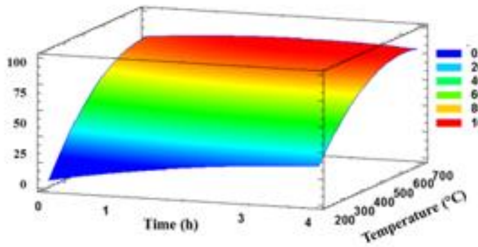

Log P = 5.09
(Octane)

Optimum values

Time (h)	Temperature (°C)
2.6	653

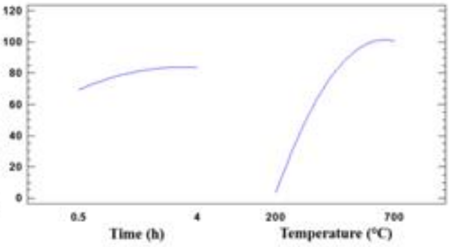

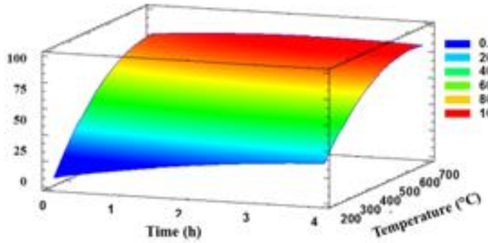

Log P = 4.47
(Heptane)

Optimum values

Time (h)	Temperature (°C)
2.7	662

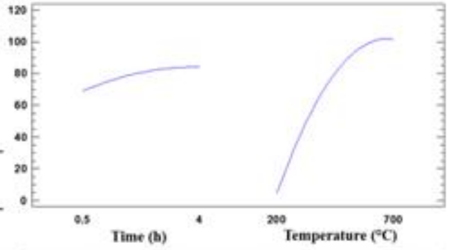

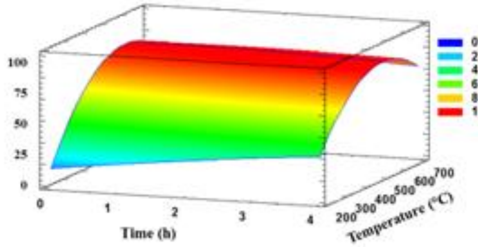

Log P = 4.09
(Decanal)

Optimum values

Time (h)	Temperature (°C)
3.1	536

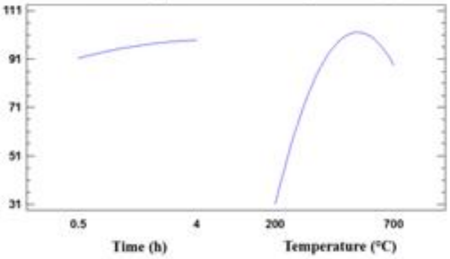

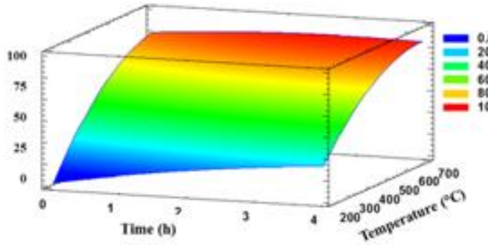

Log P = 3.39
(Cyclohexane)

Optimum values

Time (h)	Temperature (°C)
2.8	700

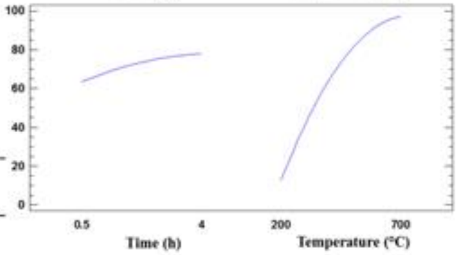

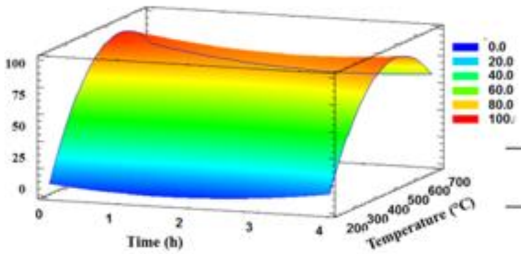

Log P = 3.03
(Octanal)

Optimum values

Time (h)	Temperature (°C)
0.5	539

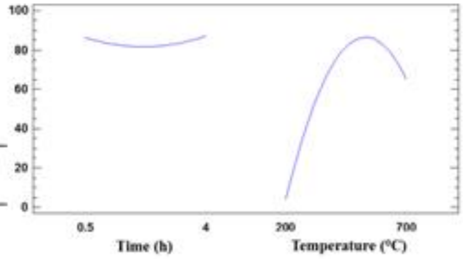

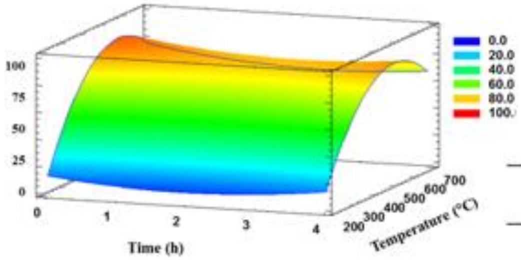

Log P = 2.50
(Heptanal)

Optimum values

Time (h)	Temperature (°C)
0.5	547

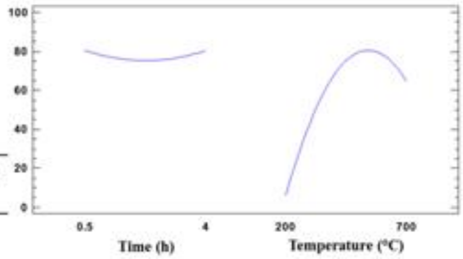

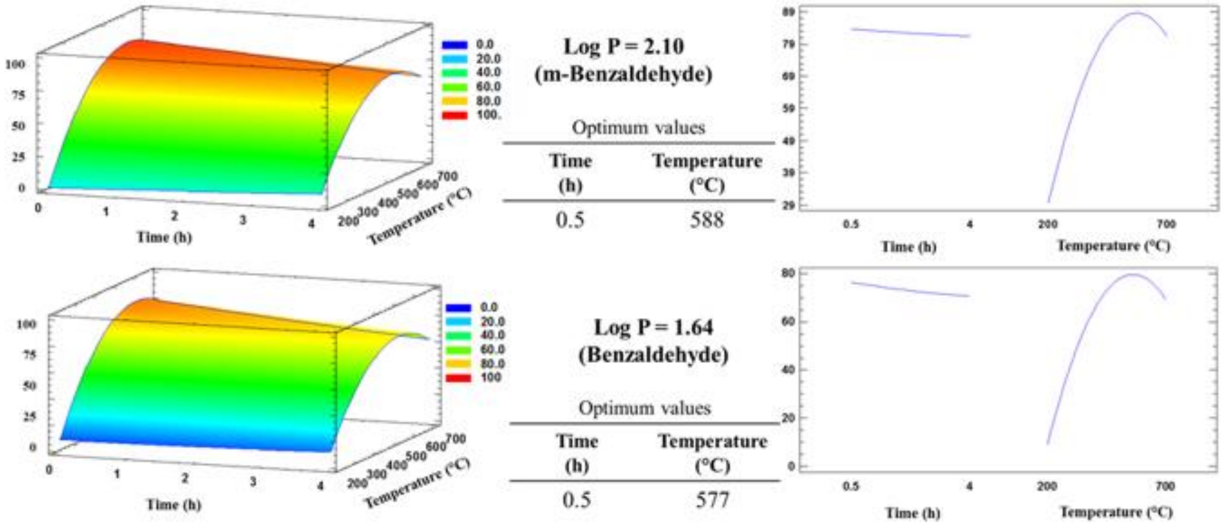

Figure S3. Surface response plot, optimum conditions and effects of the time and temperature on the synthesis of the materials for individual extraction of all studied compounds.

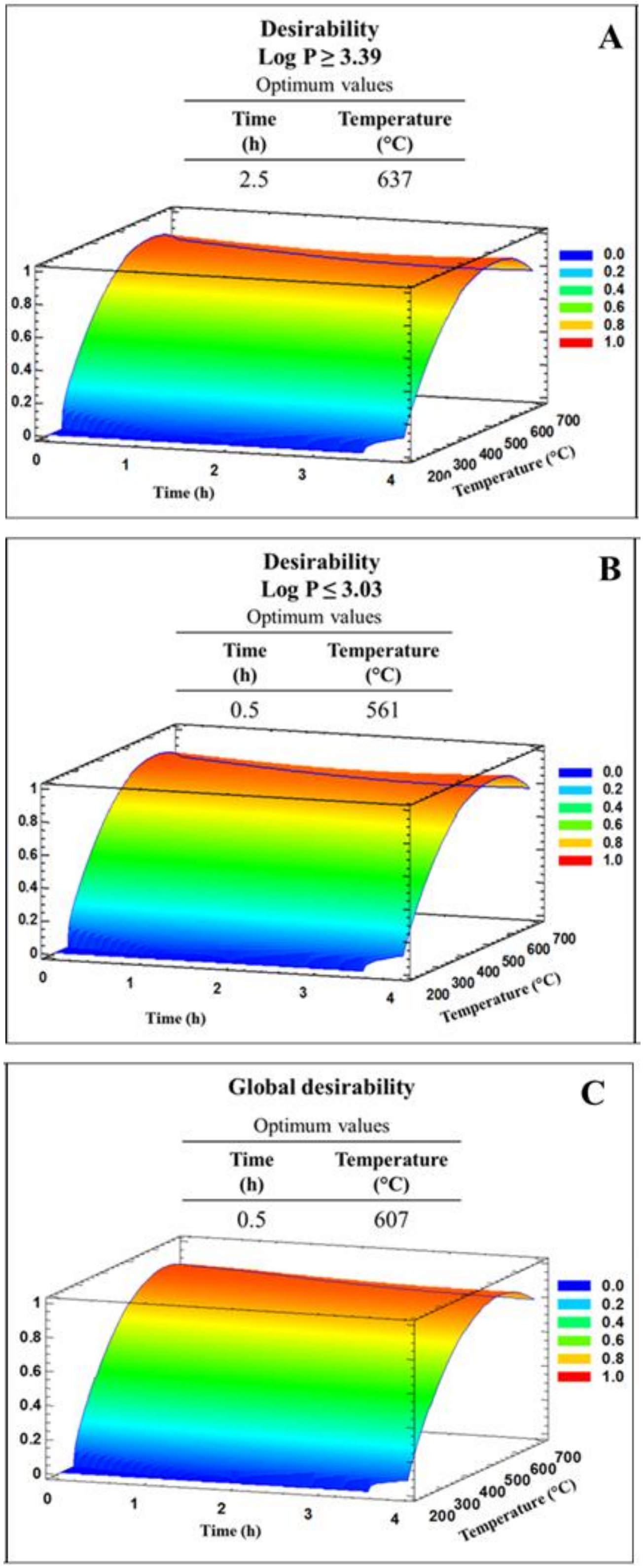

Figure S4. Surface response plot and optimum conditions for the simultaneous extraction of the (A) less polar, (B) most polar, and (C) total studied compounds.

5. Use of CFs as sorbent in analytical approaches

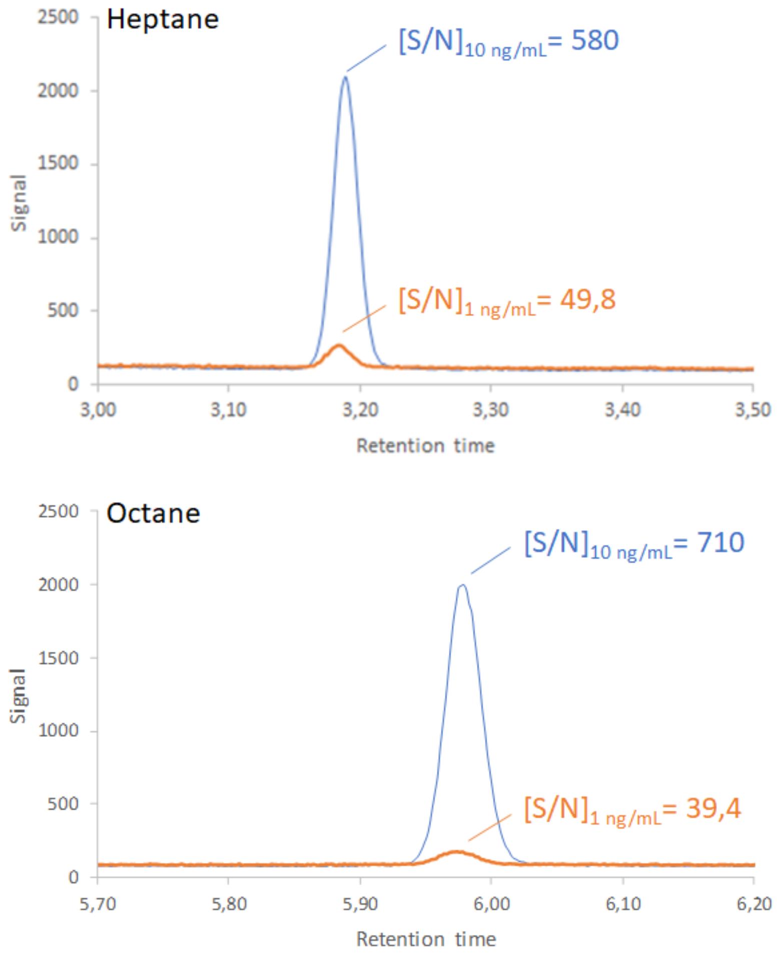

Figure S5. Detail of the chromatographic peaks obtained for heptane and octane at 10 ng/mL and 1 ng/mL. The signal to noise ratio (S/N) is also indicated.
